# Outcomes of Anterior Cruciate Ligament Reconstruction With Independently Tensioned Suture Tape Augmentation at 5-Year Follow-up

**DOI:** 10.1177/03635465231207623

**Published:** 2023-11-17

**Authors:** William T. Wilson, Matthew J. Kennedy, Douglas MacLeod, Graeme P. Hopper, Gordon M. MacKay

**Affiliations:** † Department of Biomedical Engineering, University of Strathclyde, Glasgow, UK; ‡ Department of Orthopaedics, NHS Ayrshire & Arran, Glasgow, UK; § Department of Orthopaedics, NHS Greater Glasgow & Clyde, Glasgow, UK; ‖ Department of Orthopaedics, NHS Lanarkshire, Glasgow, UK; ¶ Rosshall Hospital, Glasgow, UK; Investigation performed at Rosshall Hospital, Glasgow, UK

**Keywords:** anterior cruciate ligament, augmentation, internal brace, reconstruction, suture tape

## Abstract

**Background:**

Reconstruction using autograft remains the gold standard surgical treatment for anterior cruciate ligament (ACL) injuries. However, up to 10% to 15% of patients will suffer a graft failure in the future. Cadaveric studies have demonstrated that the addition of suture tape augmentation to ACL autograft constructs can increase graft strength and reduce elongation under cyclical loading.

**Purpose/Hypothesis:**

This study aimed to investigate the clinical outcomes and rerupture rates after ACL reconstruction (ACLR) with suture tape augmentation. We hypothesized that augmentation with suture tape would lead to lower rerupture rates.

**Study Design:**

Case series; Level of evidence, 4.

**Methods:**

Patients undergoing primary ACLR using hamstring or patellar tendon autografts augmented with suture tape between 2015 and 2019 were recruited prospectively. Patients with multiligament injuries or a concomitant lateral extra-articular procedure were excluded. Patients were observed in person for 6 months, and patient-reported outcome measures (PROMs) were collected at 2 and 5 years postoperatively. All patients were contacted, and records were reviewed to determine the incidence of graft failure. PROMs collected were as follows: Knee injury and Osteoarthritis Outcome Score (KOOS), Veterans RAND 12-Item Health Survey (VR-12), Tegner and Marx activity scores, and visual analog scale for pain (VAS).

**Results:**

A total of 97 patients, with a mean age of 34.7 (±13.4) years, were included (76% men; 52 hamstring and 45 patellar tendon grafts). The mean graft diameter was 8 (±1) mm. There was 1 rerupture (1.1%) out of the 90 patients who were contactable at a mean of 5 years postoperatively. Median KOOS scores at 2 years were as follows: Pain, 94; Symptoms, 86; Activities of Daily Living, 99; Sport and Recreation, 82; and Quality of Life, 81. The postoperative scores were significantly higher than the preoperative scores (*P* < .001). The VR-12 Physical score improved from 43 preoperatively to 55 at 2 years and remained at 56 at 5 years. The VAS pain, Tegner, and Marx scores were 0, 6, and 9, respectively, at 2 years postoperatively. There was no difference in PROMs between graft types.

**Conclusion:**

This study demonstrates encouraging results of suture tape augmentation of autograft ACLR for both hamstring and patellar tendon grafts. The failure rate of 1.1% at a mean follow-up of 5 years is lower than published rates for reconstruction, and PROMs results are satisfactory. The technique is safe to use and may permit a return to the preinjury sporting level with a lower chance of reinjury.

Injury to the anterior cruciate ligament (ACL) most commonly occurs during sports, and the incidence is increasing.^1,44,60^ Women are at higher risk of injury and reinjury after reconstruction, which is a challenging clinical problem considering the increasing number of women involved in team sports.^17,38,44,52^ Reconstruction using autograft remains the gold standard surgical treatment. However, with revision rates^12,15,43,45^ of approximately 10% to 17% and even higher in women returning to sports,^14^ the orthopaedic community strives to develop ways to make grafts as strong and resistant to failure as possible. In addition, there is a need to provide a solution that offers longevity at a high level of function, as presently only approximately 60% of patients return to their preinjury sporting level.^4^ The highest risk period for rerupture is within the first 9 months postoperatively and often occurs when athletes return to high-level activity, perhaps before graft incorporation or maturation.^25^

Recently, the concept of adding high-strength suture tape to protect and enhance the mechanical strength of biological tissue repairs^26-28^ has been employed with success in several anatomic regions, including the knee and ankle.^#^ When used with ACL reconstruction (ACLR), this augmentation theoretically protects the graft during maturation, permitting accelerated rehabilitation, and may resist reinjury upon return to sports.

Biomechanical studies have shown that adding suture tape reinforcement to a reconstruction significantly increases the ultimate tensile strength of the construct without stress shielding and reduces graft elongation.^6,7,29,53^ This added strength may reduce ACL failure rates, particularly when the graft is most vulnerable. In addition, the greatest biomechanical effect has been demonstrated with small-diameter grafts.^7^ This might permit less autograft donor tissue harvesting, potentially reducing pain in the region of the donor site and chronic weakness, which has previously been associated with graft harvest.^3,24,39,59^

Limited clinical outcome evidence is available, with small cohort studies reporting short-term follow-up data. Bodendorfer et al^9^ demonstrated improved patient-reported outcome measures (PROMs), less pain, and a higher percentage of an earlier return to preinjury activity level with suture tape augmentation. Shantanu et al^46^ showed improvements in knee laxity examination, while Parkes et al^37^ reported no difference in PROMs but higher Tegner scores in suture tape augmentation compared with conventional ACLR.

In this study, we report the clinical outcomes of patients who underwent ACLR reinforced with suture tape augmentation at a mean follow-up of 5 years postoperatively. We hypothesized that ACLR with suture tape augmentation would result in a low failure rate compared with reported rates for conventional ACLR.

## Methods

### Patient Recruitment

All consecutive patients undergoing primary ACLR using hamstring or patellar tendon autografts augmented with suture tape were included. Patients were recruited prospectively between 2015 and 2019 in a single surgeon's practice (G.M.M.), where this technique was used as standard treatment. Patients with injury to another knee ligament requiring intervention or those with a concomitant lateral extra-articular procedure were excluded. Evidence supporting the lateral extra-articular procedure with ACLR was emerging toward the end of the recruitment period. As such, exclusions on these grounds were only relevant for the final year of recruitment, in which 10 patients were excluded for young age and desire to return to professional pivoting sports. Informed consent was obtained from the participants, and ethical approval was also acquired from the local review board and ethics committee for the study. The patients underwent surgery using one of the techniques described here and followed routine clinical care pathways. Autograft selection was based on several factors, including patient preference, individual patient factors, and evolving indications.

### Surgical Technique

**Figure 1.**
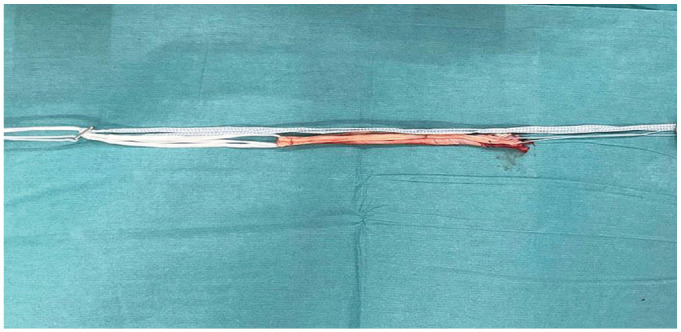
Clinical photograph showing a hamstring tendon autograft construct augmented with suture tape.

Patients were prepared and positioned in standard fashion for ACLR. Meniscal work was performed as appropriate. For hamstring autografts, a single semitendinosus tendon was harvested and prepared in either a 2-strand or a 3-strand form to produce a ≥6 mm–diameter graft (Figure 1). After transtibial tunnel drilling, the graft was passed along with the doubled 2-mm suture tape reinforcement (FiberTape; Arthrex). The proximal end was fixed using cortical suspensory fixation (RetroButton; Arthrex), with the suture tape incorporated into the button. The suture tape was then independently fixed distally, with the knee in extension. After predrilling 1 cm distal to the tibial tunnel, a 4.75-mm anchor (SwiveLock; Arthrex) loaded with both ends of the tape was placed, paying attention to avoid overtensioning. After cycling of the knee, the graft was tensioned and secured distally in the tibia using an interference screw (Biocomposite; Arthrex).

**Figure 2.**
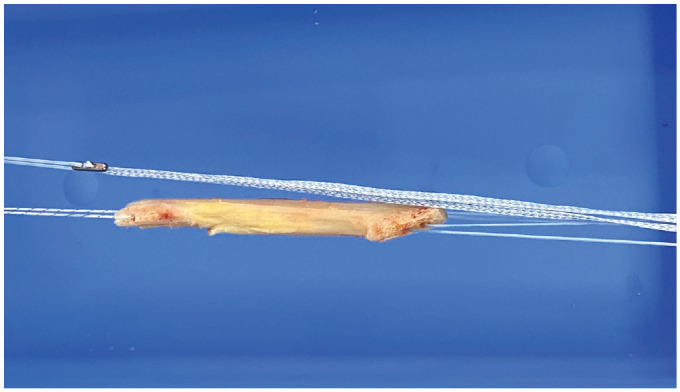
Clinical photograph showing a bone–patellar tendon–bone autograft construct augmented with suture tape.

For the patellar tendon autograft, a reduced-size graft was harvested using a standard open technique, aiming for an approximately 7- to 8-mm graft and bone block diameter^8^ (Figure 2). Tunnels were prepared similarly, and the graft was passed along with the suture tape (FiberTape), attached proximally to a button (RetroButton). An interference screw (Biocomposite) was used to fix the bone block in the femoral tunnel before the suture tape was secured distally, as described previously. Finally, with the knee in 30° of flexion, an interference screw (Biocomposite) was used to fix the distal bone block in the tibial tunnel.

Postoperatively, patients were allowed to bear weight immediately with no external splintage. All patients followed a standard physical therapy–led ACLR criterion-based rehabilitation protocol.^22^

### Follow-up

All patients were prospectively observed in person for 6 months, and PROMs were collected for ≥2 years. At the study endpoint, attempts were made to contact all patients by email, telephone, and mail, and records were reviewed to determine the incidence of graft failure based on the need for revision surgery or radiographic evidence of graft rerupture. Indications for reimaging were patients reporting instability symptoms or a further injury. PROMs were collected using the Surgical Outcomes System (Arthrex) and included the Knee injury and Osteoarthritis Outcome Score (KOOS), the Veterans RAND 12-Item Health Survey (VR-12) Physical and Mental domains, Tegner and Marx activity scores, and the visual analog scale for pain (VAS).

### Statistical Analysis

The primary outcome measure was the rerupture rate based on revision surgery or radiographic confirmation of graft failure, with secondary outcomes assessing PROM data. Data were analyzed using SPSS (IBM, Version 28), with descriptive data presented as mean (±SD) for normally distributed parameters or median (range) for nonnormal data. Group statistics were compared using independent *t* tests or Mann-Whitney tests, as appropriate for normality. Tests of significance were set at an alpha level of .05.

## Results

A total of 97 patients, with a mean age of 34.7 (±13.4) years, were included (76% men). The mean follow-up time was 5 (±1) years (range, 3.4-7.2 years). A total of 7 patients out of 97 were unable to be contacted at the time of the final review and were determined to be lost to follow-up, giving a final follow-up rate of 93%. The median time interval from injury to surgery was 6 months (range, 2 weeks–20 years).

The autologous graft types included 52 hamstring and 45 patellar tendon grafts. The mean overall graft diameter was 7.7 (±1) mm. For hamstring grafts, the mean diameter of 7 (±1) mm (range, 6-9 mm) was lower than that of the patellar tendon, which was 8.3 (±0.6) mm (range, 7-9 mm) (*P* < .001). On average, the patellar tendon group was younger than the hamstring group (31.7 [±12.3] years vs 37.4 [±13.8] years; *P* = .04). Also, the patellar tendon group had a significantly higher Tegner activity level preinjury (7 [±1] vs 6 [±2]; *P* = .01).

The most common modes of injury were soccer (48%) and skiing (23%). Meniscal pathology was addressed at the same time as ACLR in 66% of cases. Of these, 60% were lateral meniscal tears, of which 44% were repaired, while 22% of medial meniscal tears were repaired.

A single rerupture was identified in the patellar tendon group. This was in an adolescent martial arts athlete 6 months postoperatively who had returned to the sport. There were no reruptures in the hamstring group. This resulted in an overall failure rate of 1.1% at a mean 5 years postoperatively.

**Figure 3.**
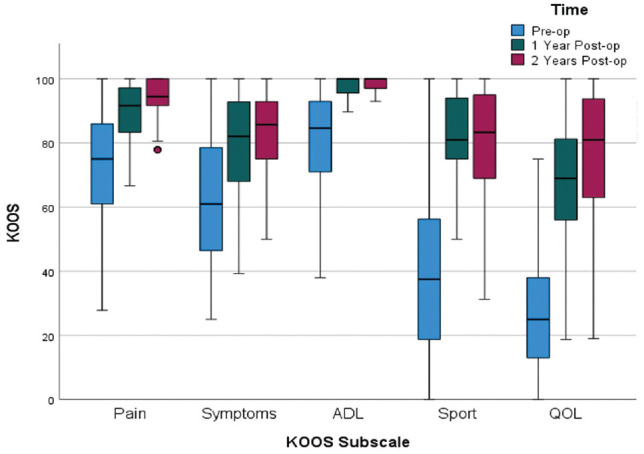
Clustered boxplot demonstrating improvement in KOOS domain scores over time. ADL, Activities of Daily Living; KOOS, Knee injury and Osteoarthritis Outcome Score; Post-op, postoperative; Pre-op, preoperative; QOL, Quality of Life; Sport, Sport and Recreation.

Median KOOS scores at 2 years were as follows: Pain, 94; Symptoms, 86; Activities of Daily Living, 99; Sport and Recreation, 82; and Quality of Life, 81. These were significantly higher than the preoperative scores (*P* < .001) for all domains (Figure 3). The median VR-12 Physical score improved from 43 preoperatively to 55 at 2 years and remained at 56 at 5 years. VAS pain scores improved overall, from a median of 2 preoperatively to a median of 0 at 2 years postoperatively. There was no difference in PROMs between graft types.

The overall mean activity scores were 6 for Tegner and 9 for Marx scores at 2 years postoperatively. There was a decrease in the Tegner score overall and for both graft types from preinjury to current level (*P* < .05). The Tegner score was lower in the hamstring group compared with the patellar tendon group, both preinjury (*P* = .01) and current (*P* < .001) (Table 1).

**Table 1:** Demographic Data and Group Characteristics^*a*^

	Hamstring	Patellar Tendon	Overall
n	52	45	97
Age, y	37.4 (±13.8)	31.7 (±12.3)	34.7 (±13.4)
Time postop at follow-up, y	5.7 (±0.7)	4.2 (±0.6)	5 (±1)
Interval injury to surgery, mo	6 (0.5-240)	6 (0.5-240)	6 (0.5-240)
Male:female, n	38:14	36:9	74:23
Graft diameter, mm	7 (±1)	8.3 (±0.6)	7.7 (±1)
Follow-up rate, %	92	93	93
Tegner preinjury	6 (±2)	7 (±1)	7 (±2)
Tegner current	5 (±2)	7 (±2)	6 (±2)

a Data are presented as mean (±SD) or median (range), unless otherwise indicated. Postop, postoperative.

A total of 12 (12%) further operations were performed in this cohort during the study period, all of which were for meniscal or chondral injuries, except for 1 revision ACLR. There were no hardware-related causes for reoperation.

## Discussion

The main finding of this study is a low failure rate of 1.1% at a mean 5 years after ACLR with independently tensioned suture tape augmentation. This is lower than the published rates for reconstruction, which range^13,15,43,45,50^ widely from 3% to approximately 25%. Some extensive registry data studies have shown that rerupture rates after reconstruction can be as low as 3% to 5% at 5 years postoperatively.^2,23^ However, 2 recent meta-analyses showed an ipsilateral reinjury rate of 7% at 2 years and up to 23% in patients who were <25 years and returning to sports.^54,58^ In this cohort, 26% of patients were <25 years old, and the rerupture rate for that subgroup was 4%. Overall, in our active group with a mean age of 34 years, a failure rate as low as 1% at 5 years postoperatively represents a successful outcome for this technique.

We compared these results with the published data from our cohort of conventional ACLR patients using a similar technique but without suture tape augmentation.^20^ That group of 272 patients had 32 reruptures (11.8%) at a mean of 5 years postoperatively. When ACLR was augmented with suture tape, the failure rate was significantly lower than reconstruction alone (χ^2^(1) = 10.1; *P* = .001). This represents a significant finding, suggesting that suture tape augmentation should be considered, particularly in high-risk patients undergoing ACLR, to increase the construct strength and reduce reinjury rates. This was not a matched comparison; the mean age was significantly younger in the group without suture tape augmentation, while the group with suture tape had higher activity scores.

Laboratory studies have shown the suture tape–augmented graft construct to be biomechanically stronger.^5-7,32,35,47,53^ Bachmaier et al^7^ showed significantly reduced elongation and higher ultimate failure load without stress shielding the hamstring graft. Wicks et al^53^ demonstrated a 33% decrease in cyclic displacement and a 25% increase in yield strength with suture tape reinforcement without increasing graft construct stiffness. Smith et al^47^ had similar findings when suture tape was used with bone–patellar tendon–bone graft constructs.

The mean graft diameter in our cohort was 8 mm for the patellar tendon group and 7 mm for the hamstring group. A hamstring autograft diameter of <8 mm has been associated with an increased risk of graft failure.^11,30,31,49^ However, despite many of the hamstring grafts in this cohort having a smaller diameter, augmenting with suture tape was associated with no graft ruptures. Bachmaier et al^7^ demonstrated that the greatest biomechanical advantages when utilizing suture tape augmentation were observed with smaller diameter hamstring grafts. Our findings add to this evidence that supports the use of suture tape as an option for a surgeon encountering a small autograft, potentially reducing intraoperative failure risk. The results also suggest that it is reasonable for surgeons to harvest smaller diameter autografts when reinforcing with suture tape, particularly with patellar tendon harvest where there is potential to reduce donor-site morbidity such as fracture, anterior knee pain, and knee extension weakness.

The single rerupture observed in our cohort was in an adolescent female patient who suffered a traumatic injury upon returning to martial arts at 6 months postoperatively. This patient underwent successful revision ACLR, which was not complicated by the presence of previous suture tape augmentation.

The higher Tegner scores observed both pre- and postoperatively for the patellar tendon group, compared with the hamstring group, are in keeping with current practice on graft choice selection based on future sporting aspirations. Although it may be associated with increased donor-site morbidity, current practice advocates the use of patellar tendon graft in high-performance athletes who will place extra demand on the biomechanical strength of the graft, which may incorporate faster.^16^ Our data demonstrate that both compare favorably in terms of rerupture, and the most significant contribution of the suture tape augmentation may be during the early phase when it acts as a “safety belt.” This may protect a hamstring graft while it incorporates and therefore improves some of its shortfalls, compared with a patellar tendon graft.

PROMS in this patient cohort compare favorably with those in the current literature on ACLR.^21,42^ The figures exceed the threshold for a Patient Acceptable Symptom State after ACLR.^34^ The small overall decrease in mean activity scores from preinjury levels observed has also been reported for patients undergoing ACLR in other studies.^36,48^

Based on our results, augmentation with suture tape is noninferior and has acceptable PROMs compared with historical conventional reconstruction. There was no incidence of reoperation for hardware irritation. This, coupled with a very low failure rate of 1.1%, represents encouraging findings for this novel technique. Our findings support the limited available clinical data on this topic, which has demonstrated a mix of equivocal and improved results for suture tape augmentation in terms of PROMs and failure rates.^9,29,37,40,46^ Our study is the first to present medium-term follow-up data, and despite a longer follow-up, demonstrates a low failure rate.

We recognize the limitations of our results being a single-surgeon, single-center practice. We have not conducted a direct matched comparison or any randomization, although we have compared it with our previously published data on conventional reconstruction. The age and activity profile of our cohort may have contributed to the low rerupture rate, and the results might not be applicable to all patient populations. Additionally, patients were not all reimaged or reexamined at the time of the study review. Furthermore, a small number of high-risk patients were excluded from enrollment in this study toward the end of the recruitment period because of the addition of the lateral extra-articular procedure to ACLR.

Given the encouraging findings demonstrated in our data, we recommend further investigation into the augmentation of ACLR with suture tape by a randomized study, adequately powered to assess differences in failure rate.

## Conclusion

This study demonstrates encouraging results of independently tensioned suture tape augmentation of autograft ACLR for both hamstring and patellar tendon grafts. The failure rate of 1.1% at a mean 5 years is lower than the published rates for ACLR, and PROM results are satisfactory. This interesting finding has the potential to improve success rates for patients returning to sports with a lower chance of reinjury. The technique also provides an option to augment smaller diameter autografts, which are at the highest risk of failure, while potentially reducing donor-site morbidity.

## Notes

# References 10, 18, 19, 26, 33, 41, 51, 55-57.
